# The amyloid-β degradation intermediate Aβ34 is pericyte-associated and reduced in brain capillaries of patients with Alzheimer’s disease

**DOI:** 10.1186/s40478-019-0846-8

**Published:** 2019-12-03

**Authors:** Tunahan Kirabali, Serena Rigotti, Alessandro Siccoli, Filip Liebsch, Adeola Shobo, Christoph Hock, Roger M. Nitsch, Gerhard Multhaup, Luka Kulic

**Affiliations:** 1 Institute for Regenerative Medicine, University of Zurich, 8952 Schlieren, Switzerland; 2 Present Address: Department of Chemistry University of Cologne, Institute of Biochemistry, 50674 Cologne, Germany; 3 Department of Pharmacology & Therapeutics and Integrated Program in Neuroscience, McGill University, Montreal, QC H3G 1Y6 Canada; 4 Neurimmune, 8952 Schlieren, Switzerland; 5 F. Hoffmann-La Roche Ltd., Roche Pharma Research & Early Development, 4070 Basel, Switzerland

**Keywords:** Alzheimer’s disease, Pericyte, Aβ34, Amyloid clearance

## Abstract

An impairment of amyloid β-peptide (Aβ) clearance is suggested to play a key role in the pathogenesis of sporadic Alzheimer’s disease (AD). Amyloid degradation is mediated by various mechanisms including fragmentation by enzymes like neprilysin, matrix metalloproteinases (MMPs) and a recently identified amyloidolytic activity of β-site amyloid precursor protein cleaving enzyme 1 (BACE1). BACE1 cleavage of Aβ40 and Aβ42 results in the formation of a common Aβ34 intermediate which was found elevated in cerebrospinal fluid levels of patients at the earliest disease stages. To further investigate the role of Aβ34 as a marker for amyloid clearance in AD, we performed a systematic and comprehensive analysis of Aβ34 immunoreactivity in hippocampal and cortical post-mortem brain tissue from AD patients and non-demented elderly individuals. In early Braak stages, Aβ34 was predominantly detectable in a subset of brain capillaries associated with pericytes, while in later disease stages, in clinically diagnosed AD, this pericyte-associated Aβ34 immunoreactivity was largely lost. Aβ34 was also detected in isolated human cortical microvessels associated with brain pericytes and its levels correlated with Aβ40, but not with Aβ42 levels. Moreover, a significantly decreased Aβ34/Aβ40 ratio was observed in microvessels from AD patients in comparison to non-demented controls suggesting a reduced proteolytic degradation of Aβ40 to Aβ34 in AD. In line with the hypothesis that pericytes at the neurovascular unit are major producers of Aβ34, biochemical studies in cultured human primary pericytes revealed a time and dose dependent increase of Aβ34 levels upon treatment with recombinant Aβ40 peptides while Aβ34 production was impaired when Aβ40 uptake was reduced or BACE1 activity was inhibited. Collectively, our findings indicate that Aβ34 is generated by a novel BACE1-mediated Aβ clearance pathway in pericytes of brain capillaries. As amyloid clearance is significantly reduced in AD, impairment of this pathway might be a major driver of the pathogenesis in sporadic AD.

## Introduction

Amyloid beta (Aβ) plaques with extracellular fibrillar deposits of Aβ peptide and neurofibrillary tangles (NFTs) composed of phosphorylated tau represent the key histopathological hallmarks of Alzheimer’s disease [22, 33, 58]. Aβ peptides are generated via sequential proteolytic cleavage of the amyloid precursor protein (APP) [47]. Initial cleavage of APP by β-secretase generates a membrane-bound C-terminal fragment of APP (β-CTF) [14, 66] which is sequentially processed by γ-secretase resulting in the formation of longer species, like Aβ40 and Aβ42/43, in addition to several other C-terminally truncated Aβ variants [39, 56]. A full set of Aβ isoforms with both N- and C-terminally truncated Aβ species can be detected in the human brain and cerebrospinal fluid (CSF) [28, 41, 42, 45, 62]. N-terminally truncated Aβ isoforms show an increased aggregation propensity and have previously been associated with the deposition of dense-core amyloid plaques in the brain [18, 35, 40, 46, 49, 51]. In contrast, C-terminally truncated shorter Aβ isoforms show a higher solubility in vitro and are more commonly detected in the cerebral vasculature [11, 18, 28, 43]. One of the C-terminally truncated isoforms, Aβ34, can be generated as an intermediate of multiple degradation pathways including Aβ degradation by the matrix metalloproteases (MMP), MMP2 and MMP9 [25], as well as stepwise proteolytic cleavage of both Aβ40 and Aβ42 by γ-secretase [39]. In addition to its role as the major β-secretase in APP processing, BACE1 (beta-site amyloid precursor protein cleaving enzyme 1) has been shown to cleave longer Aβ isoforms at position 34 and this pathway has been identified as the major source of Aβ34 [19, 50]. This additional BACE1 mediated cleavage can only take place with Aβ peptides as substrates implying that BACE1 indeed acts as an Aβ40/42 degrading enzyme to generate Aβ34 [19, 50]. In a recent study, we were able to show that Aβ34 levels are directly affected by over-expression or inhibition of BACE1 both in vitro and in vivo, and that cerebral BACE1 is the critical factor for Aβ34 generation [30]. Supporting its proposed role as a biomarker for clearance in sporadic AD, CSF Aβ34 levels were found to be elevated in subjects with mild cognitive impairment (MCI) who later converted to AD dementia [30]. Moreover, we also discovered a positive correlation between CSF Aβ34 levels and overall Aβ clearance rates in individuals with biomarker evidence of cerebral amyloid deposition [30].

In the present study, we investigated the tissue distribution of the C-terminally truncated isoform Aβ34 in post-mortem brain samples from healthy elderly individuals and AD patients of various AD neuropathological stages according to Braak [8, 9]. Aβ34 immunoreactivity was detected exclusively in the vasculature and was most prominent in brain capillaries of healthy elderly individuals in the early Braak stages. In AD patients and more advanced Braak stages, however, this capillary associated Aβ34 immunoreactivity was largely lost. Colocalization studies in postmortem brain tissue and isolated human microvessels revealed a unique association of Aβ34 with the platelet derived growth factor receptor beta (PDGFR-β) which serves as a marker for brain pericytes [1, 31]. Further in vitro biochemical studies in cultured human primary pericytes confirmed that the C-terminally truncated isoform Aβ34 is released by these cells via BACE1 mediated enzymatic cleavage of Aβ40. Collectively, the results of our work suggest the existence of a novel pericyte mediated Aβ clearance pathway, which generates Aβ34 as a stable intermediate. Early impairment of this pathway may contribute to the overall reduced Aβ clearance associated with sporadic AD [34].

## Materials and methods

### Post-mortem human samples

Paraffin-embedded hippocampus and middle-frontal cortex (gyrus frontalis medius) tissue blocks and frozen middle frontal cortex samples were provided by the Netherlands Brain Bank (NBB), the Netherlands Institute for Neuroscience, Amsterdam, the Netherlands. Post-mortem samples were collected from donors with a written informed consent for a brain autopsy and the use of the material for research purposes was obtained by the NBB. Subjects’ demographics, neuropathological and clinical evaluations are listed in Table 1. In brief, subjects are classified according to their Braak stages from stage I to VI [8, 9]. Age, gender, apolipoprotein E (APOE) genotype, CERAD (Consortium to Establish a Registry for Alzheimer’s Disease) score for amyloid load and clinical diagnosis (non-demented control (NDCNTRL) or AD patient (AD)) are provided for each subject [27, 36].

**Table 1:** Characteristics of post-mortem human brain samples

Brain Region: Hippocampus (paraffin-embedded)						
Braak Stage	Number of Subjects	Age at Death^a^	Gender^b^	APOE4 Status^c^	CERAD Score^d^	Diagnosis^e^
I	6	79 ± 6.8	M (1) F (5)	+ (1), − (2)	O(2), A(2), B(2)	NDCNTRL
II	6	85.7 ± 1.8	M (1) F (5)	+ (2), − (4)	A(1), B(3), C(2)	NDCNTRL
III	6	82 ± 5.8	M (5) F (1)	+ (3), − (2)	A(1), B(2), C(3)	NDCNTRL
IV	7	92.3 ± 3.3	M (1) F (6)	+ (2), − (5)	C	NDCNTRL (2), AD(5)
V	4	82.3 ± 10.1	M (0) F (4)	+ (3), − (1)	C	AD
VI	6	86 ± 6.8	M (0) F (6)	+ (4), − (2)	C	AD
Brain Region: Cortex (paraffin-embedded)						
Braak Stage	Number of Subjects	Age at Death	Gender	APOE4 Status	CERAD Score	Diagnosis
I	4	82.5 ± 9.3	M (2) F (2)	+ (0), − (4)	A(1), B(2), C(1)	NDCNTRL
II	3	85.3 ± 0.5	M (1) F (2)	+ (1), − (2)	A(1), B(3), C(2)	NDCNTRL
III	4	87 ± 3.7	M (3) F (1)	+ (2), − (2)	A(1), B(2), C(3)	NDCNTRL
IV	6	89.3 ± 5.4	M (2) F (4)	+ (3), − (3)	B(2), C(4)	NDCNTRL (2), AD (4)
V	3	84.3 ± 13.2	M (0) F (3)	+ (2), − (1)	C	AD
VI	5	89.2 ± 3.4	M (0) F (5)	+ (4), − (1)	C	AD
Brain Region: Cortex (frozen)						
Braak Stage	Number of Subjects	Age at Death	Gender	APOE4 Status	CERAD Score	Diagnosis
I – II	4	88.7 ± 7.9	M (0) F (4)	+ (0), − (2)	O(1), A(1), B(1), C(1)	NDCNTRL
III – IV	5	91.4 ± 2.9	M (1) F (4)	+ (1), − (2)	O(1), B(2), C(2)	NDCNTRL (3), AD (2)
V – VI	5	75.2 ± 8.1	M (2) F (3)	+ (2), −(2)	B(1), C(4)	AD

^a^ Age at death is reported as mean ± standard deviation

^b^ *M* Male, *F* Female

^c^ APOE4 Status +: APOE3/4 or APOE4/4, −:APOE3/3, APOE3/2, APOE2/2. APOE4 status were not available for some subjects

^d^ *CERAD* Consortium to Establish a Registry for Alzheimer’s Disease

^e^ *NDCNTRL* Non-demented control, *AD* Alzheimer’s disease patient

### Immunofluorescence staining

Sections of paraffin-embedded hippocampus and cortex samples with 5 μm thickness were deparaffinized in xylene and then rehydrated by immersing the slides in order in 100% ethanol, 95% ethanol, 70% ethanol and water. Sections were pretreated by boiling in 0.1 M sodium citrate buffer for antigen retrieval as previously described [20]. Only for amyloid plaque staining, sections were also incubated in 95% formic acid for 5 min prior to citrate buffer pre-treatment. Unspecific binding sites were blocked with 10% horse serum in phosphate-buffered saline (PBS) with 0.2% Triton X-100 (PBS-T). After blocking, sections were incubated overnight at 4 °C with primary antibodies diluted in 5% horse serum in PBS-T. All primary antibodies and dilutions used in immunohistochemistry are listed in Additional file 1. After washing with PBS, sections were incubated for 2 h at room temperature with secondary antibodies (1:200 dilution in 5% horse serum in PBS-T, donkey anti-mouse/rabbit/goat conjugated to Alexa488, Cy3, Alexa647 and streptavidin conjugated to Alexa488) purchased from Jackson Immunoresearch (Pennsylvania, USA). Following the secondary antibody step, sections were incubated for 10 min with 2 mg/ml DAPI (4′,6-diamidino-2-phenylindole) (Sigma Aldrich, Missouri, USA) in PBS for nucleus staining and for 5 min in 0.2% Sudan Black (Sigma Aldrich) in 70% ethanol to quench auto-fluorescence [38]. Coverslips were mounted with water based mounting medium and slides were stored at 4 °C. Pictures were taken with Leica DM4000B microscope or Leica TCS SP8 confocal microscope. For peptide blocking/competition assay, 1 μg anti-Aβ34 and 1 μg anti-PDGFR-β antibodies were pre-incubated with 10 μg recombinant human Aβ34 peptide (Anaspec, California, USA) in 5% horse serum in PBS-T for 1 h. The immunohistochemistry protocol was then followed as described above.

### Quantification of immunofluorescence staining

For each subject, 2 sections, which are at least 50 μm apart from each other, were used and 10 pictures with 20X magnification were randomly taken per section. For Aβ34 and PDGFR-β quantitative analyses, sections were also stained with anti-Collagen IV antibody to allow a quantification of the total number of vessels in each visual field. Vessels with Aβ34 or PDGFR-β immunoreactivity were counted manually and divided by the total number of vessels in the visual field assessed by Collagen IV immunostaining. In order to quantify only capillaries, vessels with a diameter > 10 μm were excluded. Mean of 20 visual fields were calculated for each subject and results were reported as percentage of Aβ34 or PDGFR-β positive vessels. For tau tangle and amyloid plaque quantifications, pictures were converted to 8-bit images and constant thresholds were applied to each picture. Intra-neuronal APP signal was also eliminated by using this threshold in amyloid plaque quantification. The area for each immunostained picture was calculated with ImageJ and results were reported as percentage of amyloid or tau positive area. Mean of 20 visual fields were calculated for each subject and reported in the figures.

### Microvessel isolation and preparation of vessel enriched brain lysates

Previously published microvessel isolation protocols for mouse and rat brains were adapted for frozen human brains [6, 10, 65]. Briefly, 1 g frozen human cortex was placed in 5 ml Hank’s balanced salt solution (HBSS) with protease and phosphatase inhibitors and homogenized with glass Teflon homogenizer (20 strokes at 400 rpm). Homogenates were centrifuged at 2000 g for 10 min at 4 °C. The pellet was resuspended in 17.5% Dextran (Sigma Aldrich) in PBS and centrifuged at 4400 g for 15 min at 4 °C. The pellet was again resuspended in 1 ml HBSS and passed through filters with 100 μm and 20 μm pore size (Pluriselect Life Science, Leipzig, Germany) in the respective order. The material retained on the 20 μm filter was collected by gentle washing with HBSS and centrifuged at 2000 g for 10 min at 4 °C. The resulting pellet (corresponding to microvessels) was resuspended in 4% paraformaldehyde (PFA) in PBS for fixation. Similar to the immunofluorescence protocol, microvessels were blocked with 10% horse serum in PBS-T and incubated 2 h with primary antibodies and 2 h with secondary antibodies in PCR tubes at room temperature. Microvessels were then placed on glass slides and coverslips were mounted with water-based mounting medium. Images were taken with Leica TCS SP8 confocal microscope. For vessel enriched brain lysates, material retained on both filters was collected and combined by washing with HBSS and centrifugation at 2000 g for 10 min at 4 °C. After centrifugation, the pellet was incubated in RIPA buffer at 4 °C with rotation for 1 h, sonicated for 5 min in ultrasonic bath and then centrifuged at 10000 g for 15 min at 4 °C. Supernatants were collected and stored at -80 °C.

### Immunoassays

Custom-made 4-plex (Aβ34, Aβ38, Aβ40, and Aβ42) meso scale discovery (MSD) plates were purchased from Meso Scale Diagnostics (Maryland, USA). For 1-plex Aβ34 assay, high binding MSD plates were spot-coated with monoclonal anti-Aβ34 antibody and incubated overnight. The assay was performed according to manufacturer’s instructions as previously reported [30]. Briefly, diluent 35 was added into each well and incubated for 1 h at room temperature for blocking. PBS with 0.2% Tween-20 was used for washing. 25 μl sample or standard and 25 μl sulfo-tag anti-amyloid beta (6E10 or 4G8) detection antibody were added. Plates were incubated at 4 °C overnight with rigorous shaking. On the following day, 2X read buffer was added after washing and plates were read with SECTOR Imager 600 plate reader (Meso Scale Diagnostics, USA) and analyzed with MSD Workbench software (Meso Scale Diagnostics, USA).

The human total PDGFR-β enzyme-linked immunosorbent assay (ELISA) and ELISA ancillary reagent kits were purchased from R&D Systems (Minnesota, USA). The assay was performed according manufacturer’s instructions. Briefly, plates were incubated with capture antibody diluted in PBS overnight at 4 °C. PBS with 0.2% Tween-20 was used for washing. Aliquots of 100 μl sample or standard were diluted in diluent 12 and incubated for 2 h at room temperature. After washing, biotinylated detection antibody, diluted in diluent 14, was added into wells and incubated for 2 h at room temperature. Following a wash step, streptavidin-horseradish peroxidase (HRP) substrate diluted in diluent 14 was added. Color reagents were added and incubated for 20 min; the reaction was stopped by adding stop solution (2 N sulfuric acid) and absorbance at 520 nm was measured with Tecan Infinite M Nano plate reader.

### Cell culture

Human primary pericytes were purchased from ScienCell (California, USA). Cells were maintained in Pericyte Medium (ScienCell) with addition of 2% fetal bovine serum (FBS) (ScienCell), penicillin/streptomycin (ScienCell) and pericyte growth supplement (ScienCell) in poly-L-lysine (ScienceCell or SigmaAldrich) coated T75 flasks. Cells from each passage were frozen in 10% dimethyl sulfoxide (DMSO) in FBS and stored at -150 °C. Cells from passage 3 to 6 were used in experiments.

For Aβ uptake assays, human recombinant Aβ40 (rPeptide, Watkinsville, USA) and mouse Aβ40 synthetic peptides (Anaspec, California, USA) were prepared according to published protocols [52]. Briefly, lyophilized peptides were dissolved in hexafluoro-2-propanol (HFIP) on ice and aliquoted into 1.5 ml Eppendorf tubes. Aliquots were lyophilized and stored at -80 °C. Before the experiment, lyophilized peptide was dissolved in DMSO and concentration adjusted with pericyte medium. Pericytes were cultured in either 60-mm dishes or 6-well plates and treated with different concentrations of Aβ40 for varying incubation times. At the end of the experiment cell lysates were prepared in RIPA buffer, cell media were collected and stored for further analysis. In order to block Aβ uptake, cells were treated with 10 μM endocytosis blocker IPA3 (1,1′-Dithiodi-2-naphthtol) (Abcam, Cambridge, United Kingdom) or 500 nM low density lipoprotein receptor-related protein 1 (LRP1) blocker RAP (receptor associated protein) (R&D Systems) for 2 h prior to Aβ40 (2 μM) addition. Solutions of 10 μM IPA3 and 500 nM RAP were used since these concentrations were reported to reduce Aβ40 uptake without affecting cell viability [61]. Cell lysates were prepared with RIPA buffer, cell media were collected and stored for further analysis. In order to study Aβ uptake, pericytes were seeded on coverslips in a 24-well plate and incubated with Hilyte Fluor 488-labeled human Aβ40 (Anaspec) after RAP or IPA3 treatment. After fixation and DAPI staining, cells were imaged with Leica TCS SP8 confocal microscope. For 96-well assay, pericytes were plated in 96-wells and incubated with Hilyte Fluor 488-labeled human Aβ40 after RAP or IPA3 treatment. After washing, extracellular Aβ was quenched with 0.2% trypan blue fluorescence and measured at 485 nm excitation and 535 nm emission wavelength for detection of Hilyte Fluor-488 with Tecan Spark plate reader. Subsequently, cells were incubated with DAPI and fluorescence was measured with 360 nm excitation and 485 nm emission wavelength to normalize Hilyte Fluor 488 fluorescence for cell count in each well. For BACE1 inhibition assays, pericytes were treated with varying concentrations (10 μM, 5 μM, 2 μM, 1 μM) of BACE1 Inhibitor IV (Sigma Aldrich) for 12 h prior to Aβ40 (2 μM) addition. As in previous experiments, lysates were prepared with RIPA buffer, cell media were collected and stored for further analysis.

### Immunocytochemistry

Coverslips were coated with poly-L-lysine and placed in 24-well plate. Cells were seeded in 24-well plates, incubated overnight and then fixed with 4% PFA in PBS. Unspecific binding was blocked with 10% horse serum in PBS. Cells were incubated with primary antibody for 2 h, followed by secondary antibody for 90 min at room temperature (Additional file 1). For nucleus staining, cells were incubated with 2 mg/ml DAPI solution in distilled water for 10 min. Then, coverslips were transferred on slides with water-based mounting medium. Cells were imaged with Leica DM4000B microscope or Leica TCS SP8 confocal microscope.

### Western blot and immunoprecipitation

Pericyte lysates were mixed with 2X Novex SDS sample buffer (Thermo Fisher Scientific, Massachusetts, USA) containing 10% β-mercaptoethanol 1:1, incubated at 95 °C for 5 min and loaded on Novex 10–20% Tricine gel (Thermo Fisher Scientific). Electrophoresis was performed at 100 V for 2 h in Tricine running buffer. After transfer, nitrocellulose membranes were blocked with 5% milk blocking solution and incubated with primary antibodies at 4 °C overnight. On the next day, membranes were incubated with HRP-coupled secondary antibodies (Jackson Immunoresearch) diluted in 5% milk blocking solution for 2 h at room temperature and developed with SuperSignal West Femto Maximum Sensitivity Substrate (ThermoFisher). Images were taken with ImageQuant LAS 400 (GE Healthcare, Illinois, USA). BACE1 immunoprecipitation was performed using magnetic separation with Dynabead M-280 Sheep Anti-Rabbit IgG (Invitrogen) according to the manufacturer’s protocol. Briefly, cell lysates were incubated with rabbit anti-BACE1 antibody (ab2077, abcam) overnight at 4 °C with gentle rotation. After addition of magnetic beads, samples were incubated for 1 h with gentle rotation at room temperature. After washing with PBS, beads were magnetically separated and incubated in 2X Novex SDS sample buffer containing 10% β-mercaptoethanol at 95 °C for 5 min. Beads were magnetically separated and proteins loaded on Novex 10–20% Tricine gel. Electrophoresis and Western blotting were performed as described above.

### Statistical analysis

Graphpad Prism 8 software was used for all statistical analyses. Assumption of normal (Gaussian) distribution was tested with Shapiro-Wilk normality test (α = 0.05). If the assumption of normality was true, the significance of the differences between two groups was tested with a two-tailed unpaired Student’s t test. The significance of the differences between more than two groups was tested with 1-way analysis of variance (ANOVA) followed by Tukey’s multiple comparison test. Correlations were calculated with Pearson’s correlation (α = 0.05, confidence interval 95%, two tailed). In case of a deviation from normal distribution, the significance of the differences between two groups was tested with a two-tailed unpaired Mann-Whitney test. Correlations were calculated with Spearman’s correlation (α = 0.05, confidence interval 95%, two tailed). Correlation coefficients (r) and *p*-values were reported in the figures.

## Results

### Aβ34 immunoreactivity is detected in brain capillaries of non-demented elderly individuals and this immunoreactivity is progressively reduced in AD patients

**Fig. 1:**
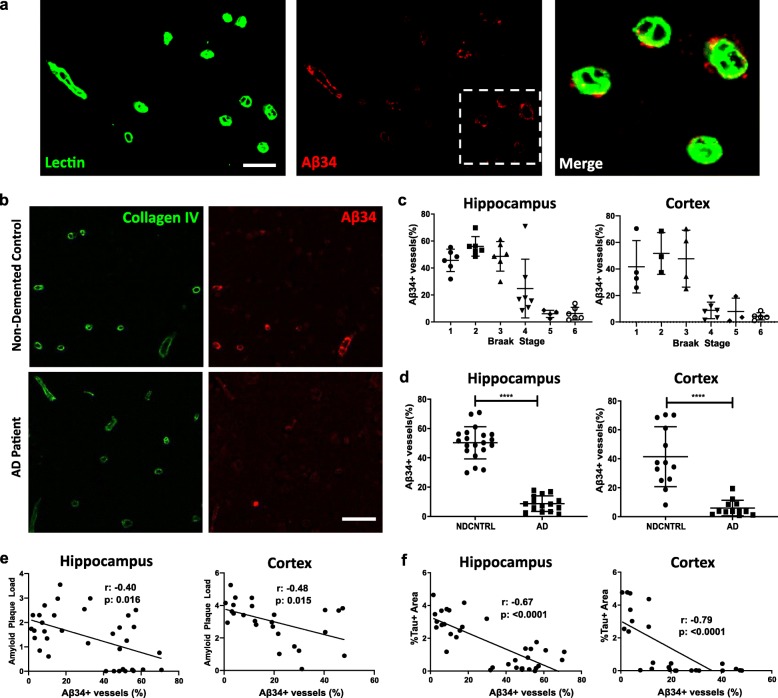
Capillary associated Aβ34 immunoreactivity is reduced in Alzheimer’s disease patients and inversely correlated with both amyloid and tau. **a** Double immunostaining of vascular marker, Lectin (green), and Aβ34 (red) in human post-mortem brain. Merged image shows magnification of the dashed area. (Scale bar 10 μm) **b** Representative images of double immunostaining of basement membrane marker, Collagen IV (green) and Aβ34 (red) in a non-demented control and an AD patient for quantification of capillary Aβ34 immunoreactivity. In each visual field number of Aβ34+ vessels divided by total number of vessels (diameter < 10 μm) (calculated with Collagen IV immunostaining) and results reported as percentage of Aβ34+ vessels. (Scale bar 10 μm) **c** Braak-stage distribution for % of Aβ34+ vessels in hippocampus and cortex. Graphs represent individual values and mean with standard deviation (SD). **d** Diagnosis-based distribution of % of Aβ34+ vessels in hippocampus and cortex. Graphs represent individual values and mean with SD. **** (*p* < 0.001) was determined by a two-tailed unpaired student’s t test. **e** Correlation of %Aβ34+ vessels with amyloid plaque load in hippocampus and cortex. Amyloid plaque load was calculated by using % area covered by immunostaining of N-terminal anti-amyloid (clone W02) antibody. For hippocampus Pearson’s correlation (α = 0.05, CI 95%, two-tailed) and for cortex Spearman’s correlation were (α = 0.05, CI 95%, two-tailed) used. **f** Correlation of %Aβ34+ vessels with tau tangles in hippocampus and cortex. Tau+ area was calculated by using % area covered by anti-tau (clone AT8) antibody. Correlations were calculated using Spearman’s correlation (α = 0.05, CI 95%, two-tailed)

In this study, we performed a comprehensive disease-stage dependent immunohistochemical analysis of Aβ34 immunoreactivity in hippocampal and cortical brain tissue from cognitively intact elderly individuals and AD patients of various Braak stages (Table 1). Throughout disease stages, Aβ34 was predominantly found in small vessels, which were identified as brain capillaries based on their structure and diameter of < 10 um (Fig. 1a, Additional file 2a). Notably, these Aβ34 positive capillaries were free of fibrillar amyloid (assessed by Thio S staining, Additional file 2b), and capillary congophilic amyloid angiopathy (CAA) was generally absent in the investigated sample. Moreover, Aβ34 was not detectable in parenchymal amyloid plaques, but was occasionally observed in colocalization with arterial CAA deposits in a minority of the assessed brain samples (Additional file 2b-c). To further quantitatively assess the predominant Aβ34 immunoreactivity in brain capillaries of AD patients and non-demented control individuals, double immunofluorescence staining with the basement membrane marker collagen IV was performed (Fig. 1b). The stainings revealed that in non-demented elderly individuals up to 60–70% of capillaries showed immunoreactivity for Aβ34 (Fig. 1d). In contrast, the proportion of Aβ34 positive capillaries in patients with AD clinical diagnosis was strongly reduced (Fig. 1d), whereas the capillary density was not significantly changed (Additional file 2d). A disease stage dependent analysis of Aβ34 immunoreactivity in the whole sample revealed a peak of Aβ34 immunoreactivity in the early Braak stages (highest percentage of Aβ34 positive capillaries in Braak stage 2) and a dramatic loss of Aβ34 positive vessels between Braak stages 3 and 4 (Fig. 1c). Interestingly, Aβ34 immunoreactivity negatively correlated both with amyloid plaque load (Fig. 1e) and with tau pathology (Fig. 1f) in hippocampus and cortex; however, stronger effects were observed for the association of Aβ34 with tau (Fig. 1).

### Aβ34 in capillaries colocalizes with pericytes which are gradually lost in advanced disease stages

**Fig. 2:**
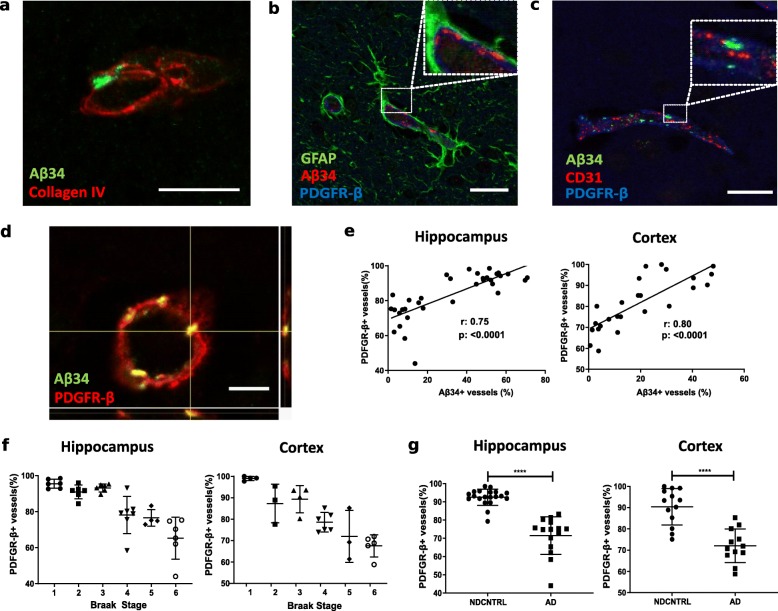
Aβ34 is associated with PDGFR-β + pericytes in small capillaries and Aβ34 immunoreactivity is strongly correlated with pericyte coverage **a** Merged image of Aβ34 (green) and basement membrane marker, Collagen IV (red) immunostaining. (Scale bar 10 μm) **b** Merged image of astrocyte marker, GFAP (green), Aβ34 (red) and pericyte marker, PDGFR-β (blue) immunostaining (Scale bar 20 μm). Insert shows magnification of the dashed area. **c** Merged image of Aβ34 (green), endothelial cell marker, CD31 (red) and pericyte marker, PDGFR-β (blue) immunostaining (Scale bar 20 μm). Insert shows magnification of the dashed area. **d** Maximum projection of confocal merged image of Aβ34 (green) and pericyte marker PDGFR-β (red). (Scale bar 5 μm). **e** Correlation of % Aβ34+ vessels with % PDGFR-β + vessels in hippocampus and cortex. % of PDGFR-β + vessels represents the number of PDGFR-β + vessels divided by total number of vessels assessed by Collagen IV immunostaining for each visual field. For hippocampus Pearson’s correlation (α = 0.05, CI 95%, two-tailed), for cortex Spearman’s correlation were (α = 0.05, CI 95%, two-tailed) used. **f** Braak stage distribution of %PDGFR-β + vessels (pericyte coverage) in hippocampus and cortex. Graphs represent mean with standard deviation (SD). **g** Diagnosis-based distribution of %PDGFR-β + vessels in hippocampus and cortex. Graphs represent individual values and mean with SD. **** (*p* < 0.001) was determined by a two-tailed unpaired Mann-Whitney test for hippocampus and student’s t-test for cortex

Having shown that Aβ34 in non-demented elderly individuals and in patients with AD was mainly associated with brain capillaries, and that the percentage of Aβ34 immunoreactive vessels was progressively reduced in advanced AD neuropathological stages, we determined next in more details the exact localization of capillary Aβ34 immunoreactivity within the neurovascular unit. Double immunofluorescence stainings with basement membrane marker Collagen IV (Fig. 2a), astroglial marker glial fibrillary acidic protein (GFAP) (Fig. 2b), endothelial cell marker CD31 (Fig. 2c) and pericyte marker PDGFR-β (Fig. 2b-c-d) were performed. These analyses demonstrated a robust overlay of Aβ34 immunoreactivity with PDGFR-β, but not with CD31, nor with GFAP, suggesting that Aβ34 was specifically localized in brain pericytes (Fig. 2b-c-d). In line with these findings, a close association was also observed with Collagen IV which represents a major extracellular component of the capillary basement membrane where pericytes reside (Fig. 2a). Given the observed colocalization of Aβ34 immunoreactivity with brain pericytes within the neurovascular unit and the above-mentioned disease-stage dependent loss of Aβ34 positive capillaries in AD, we hypothesized that the progressive decrease in Aβ34 positive capillaries in the course of AD could at least partially be explained by the previously reported dysfunction and loss of brain pericytes in AD pathogenesis [23, 48]. In agreement with this hypothesis, a strong positive correlation could be established between the percentage of Aβ34 positive and PDGFR-β immunoreactive brain capillaries (Fig. 2e). The analysis of PDGFR-β immunoreactivity across Braak stages revealed a gradual loss of pericyte coverage starting already at Braak stage 2 (Fig. 2f). Similar to our results of the quantitative assessments of Aβ34 immunoreactivity, the percentage of PDGFR-β positive capillaries was significantly decreased in AD patients in comparison to non-demented controls both in cortex and hippocampus (Fig. 2g).

### Aβ34 is present in brain pericytes from isolated human microvessels and its levels are correlated with pericyte marker PDGFR-β in non-demented controls

**Fig. 3:**
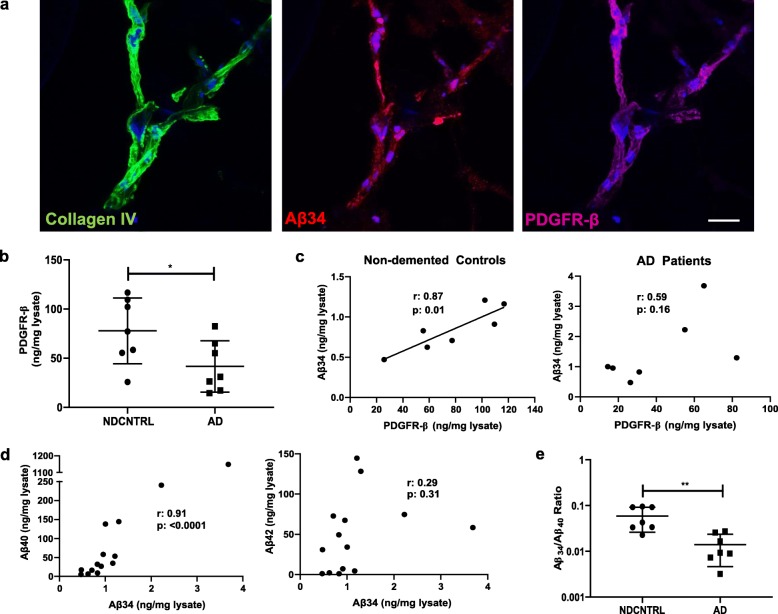
Strong association between pericytes and Aβ34 in microvessels isolated from frozen human cortex. **a** Immunostaining of basement membrane marker, Collagen IV (green), Aβ34 (red), pericyte marker, PDGFR-β (blue) and DAPI (blue) in a microvessel isolated from human cortex. (Scale bar 20 μm). **b** Distribution of PDGFR-β levels (normalized to total protein content) in vessel enriched brain lysates for non-demented controls and AD patients. Graph represent individual values and mean with SD. * (*p* < 0.05) was determined by a two-tailed unpaired student’s t test. **c** Correlation of Aβ34 and PDGFR-β levels (normalized to total protein content) in vessel enriched brain lysates in non-demented controls and AD patients. Correlations were calculated using Pearson’s correlation (α = 0.05, CI 95%, two-tailed). **d** Correlation of Aβ34 and Aβ40 or Aβ42 levels (normalized to total protein content) in vessel enriched brain lysates. For Aβ34 and Aβ40 correlation, Spearman’s correlation (α = 0.05, CI 95%, two-tailed) was used. For Aβ34 and Aβ42 correlation, Pearson’s correlation (α = 0.05, CI 95%, two-tailed) was used. **e** Distribution of Aβ34/Aβ40 ratio in vessel enriched brain lysates for non-demented controls and AD patients. Graph represent individual values and mean with SD. ** (*p* < 0.01) was determined by a two-tailed unpaired Mann-Whitney test

Collectively, our immunohistochemical findings revealed a predominant localization of Aβ34 in brain capillaries and a robust colocalization and strong correlation of Aβ34 with pericyte markers. Moreover, we observed a progressive loss of capillary Aβ34 immunoreactivity across disease stages which was paralleled by a gradual loss of pericyte coverage. To further investigate the relationship between Aβ34 and its longer precursors, Aβ40 and Aβ42, as well as pericyte markers, microvessels were isolated from frozen cortical samples of healthy non-demented controls and AD patients (Table 1). In line with the results of our immunohistochemical analysis of human postmortem brain tissue, immunofluorescence staining confirmed a colocalization of Aβ34 immunoreactivity with pericyte marker PDGFR-β and basement membrane marker Collagen IV in the isolated microvessels (Fig. 3a). Similar to the immunohistochemical findings, quantitative analysis of RIPA-buffer extracted vessel enriched brain lysates demonstrated a significant decrease in total PDGFR-β levels in AD patients in comparison to non-demented controls (Fig. 3b) and a positive correlation between total PDGFR-β and Aβ34 levels in non-demented individuals (Fig. 3c). Interestingly, a strong positive correlation was recorded between the levels of vessel-extracted Aβ34 and Aβ40, but not with Aβ42 (Fig. 3d). A significantly decreased Aβ34/Aβ40 ratio was observed in microvessels from AD patients in comparison to non-demented controls suggesting a reduced proteolytic degradation of Aβ40 to Aβ34 in AD (Fig. 3e).

### Human primary pericytes are capable of degrading Aβ40 in a BACE1-dependent manner

**Fig. 4:**
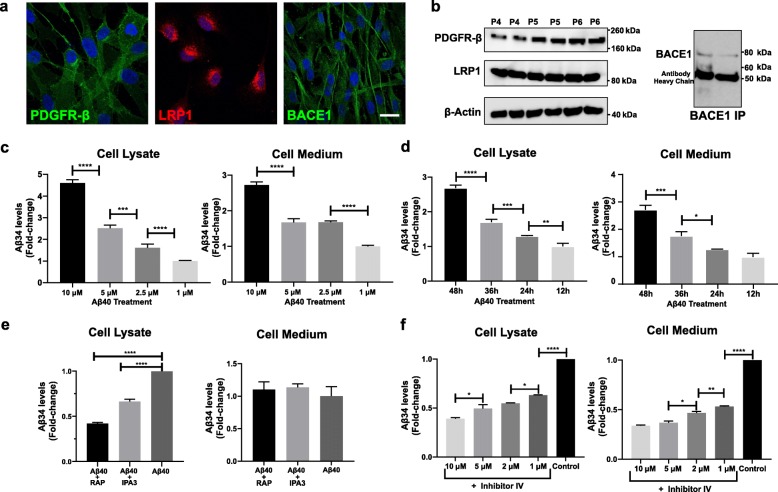
Human primary pericytes take up extracellular Aβ40 via LRP1-mediated endocytosis and generate Aβ34 via BACE1-mediated enzymatic degradation. **a** Immunostaining of human primary pericytes with PDGFR-β, LRP1, BACE1 and DAPI (blue). (Scale bar 10 μm) **b** Levels of PDGFR-β and LRP1 in pericyte lysates from different passages (passage 4 (P4), passage 5 (P5) and passage 6 (P6)) assessed by western blot. Levels of BACE1 in pericyte lysate were also assessed by western blot following immunoprecipitation (IP). **c** Aβ34 levels in cell lysate and medium following 3 days incubation with varying concentrations of human recombinant Aβ40 peptide. Aβ34 levels were normalized and shown as fold-change compared to lowest concentration of Aβ40 treatment (1 μM). **d** Aβ34 levels in cell lysate and medium following incubation with 5 μM human recombinant Aβ40 peptide for varying durations. Aβ34 levels were normalized and shown as fold-change compared to shortest duration of Aβ40 treatment (12 h). **e** Aβ34 levels in cell lysate and medium following 2 h incubation with 10 μM IPA3 or 500 nM RAP prior to 12 h incubation with 2 μM Aβ40. Aβ34 levels were normalized and shown as fold-change compared to only Aβ40 treated cells. **f** Aβ34 levels in cell lysate and medium following 12 h incubation with varying concentrations of BACE1 inhibitor (Inhibitor IV) prior to 12 h incubation with 2 μM Aβ40. Aβ34 levels were normalized and shown as fold-change compared to only Aβ40 treated cells. The graphs (**c-f**) represent the mean ± SD. **** (*p* < 0.0001), *** (*p* < 0.001), ** (*p* < 0.01) and * (*p* < 0.05) were determined by 1-way ANOVA followed by Tukey’s multiple comparison test

Based on our findings in human post-mortem brain tissue and in isolated microvessels, we hypothesized an early involvement of brain pericytes in enzymatic Aβ degradation leading to the generation of Aβ34 as an intermediate, and a failure of this clearance pathway with advancing disease stages in AD. We moreover observed a positive correlation between Aβ34 and Aβ40 in isolated human microvessels from non-demented elderly individuals suggesting that Aβ40 could be the main substrate for uptake and potential enzymatic degradation to Aβ34 at the neurovascular unit. To assess whether pericytes are capable of generating Aβ34 in vitro, uptake and degradation of extracellularly applied Aβ40 were further investigated in human primary pericyte cultures. As a first step, expression of the Aβ uptake receptor, low density lipoprotein receptor-related protein 1 (LRP1), and of the major Aβ34 producing enzyme, BACE1, were confirmed by immunocytochemistry and Western blotting (Fig. 4a-b). In the following, human primary pericytes were incubated with varying concentrations (10 μM, 5 μM, 2.5 μM and 1 μM) of recombinant human Aβ40 peptide for different treatment durations (12 h, 24 h, 36 h, 48 h). These experiments revealed a dose and time dependent increase in Aβ34 levels both in cell lysates and in cell media (Fig. 4c-d). In order to assess whether Aβ34 generation in primary pericytes was dependent on the uptake of extracellular Aβ40, cultures were treated with either a generic endocytosis blocker, IPA3, or with a specific LRP1 blocker, receptor associated protein (RAP). Treatment with both compounds significantly reduced the uptake of fluorescently-labeled Aβ40 in human primary pericytes (Additional file 3a-b). Aβ34 levels in cell lysates were significantly reduced when pericytes had been pre-incubated with IPA3 or RAP before Aβ40 treatment (Fig. 4e) thus confirming that internalization of extracellular Aβ40 is crucial for Aβ34 generation in pericytes. In order to rule out the possibility of an increased de novo synthesis of Aβ34 in pericytes upon Aβ40 treatment, we further evaluated the source of Aβ34 generated in the pericyte culture. For this purpose, pericytes were treated with mouse Aβ40 and levels of Aβ34 were measured by using either a mid-domain (clone 4G8) or an N-terminal (clone 6E10) anti-amyloid detection antibody. The N-terminal antibody had previously been shown to be human specific whereas the mid-domain antibody is known to detect both human and murine Aβ isoforms [57]. After treatment with mouse Aβ40, only the mid-domain antibody was able detect the increase in Aβ34 levels while the N-terminal, human Aβ specific, antibody failed to detect any changes (Additional file 3c). The result of this experiment therefore suggested that upon treatment with mouse Aβ40, human pericytes predominantly generated mouse Aβ34 via degradation of extracellularly applied Aβ40. Enzymatic degradation of Aβ in pericytes was further investigated upon pharmacological inhibition of the major degrading enzyme. Since Aβ34 was identified as an intermediate of BACE1-mediated enzymatic degradation and BACE1 expression could be confirmed in human primary pericyte culture, pericytes were treated with varying concentrations (10 μM, 5 μM, 2 μM and 1 μM) of the BACE1 inhibitor IV, and Aβ34 levels were assessed upon BACE 1 inhibition. Treatment of pericytes with inhibitor IV prior to Aβ40 treatment led to a dose dependent decrease in Aβ34 levels in pericytes (Fig. 4f) supporting the role of BACE1 in Aβ34 generation in pericytes. Consequently, our in vitro cell culture results demonstrate that Aβ34 can be generated as an intermediate of BACE1-mediated enzymatic degradation of internalized Aβ40 in pericytes.

## Discussion

The C-terminally truncated Aβ isoform, Aβ34, has been identified as an important intermediate product of enzymatic Aβ degradation clearance. Although, in principle, Aβ34 can be generated via various enzymatic pathways, BACE1 was recently shown to be a major enzyme involved in Aβ34 generation in vivo [30]. In our recently published study, we showed elevated CSF Aβ34 levels in early clinical stages of AD and a correlation of CSF Aβ34 levels with Aβ clearance rates in subjects with evidence of cerebral amyloid deposition [30]. In the present study, we analyzed the distribution of Aβ34 in post-mortem human brain samples to further investigate its role in AD pathology and neurodegenerative disease progression. Our comprehensive histological analysis revealed a unique association of Aβ34 immunoreactivity with pericytes in brain capillaries and a loss of this capillary associated Aβ34 immunoreactivity with advancing disease stages in AD patients. Interestingly, we observed a peak in capillary Aβ34 immunoreactivity in the earlier Braak stages (stage 2–3) which then dramatically decreased between Braak stages 3 and 4. The correlation of Braak stages with cognitive scores used in clinical practice had been investigated by several previous studies suggesting that individuals with MCI were most likely distributed between Braak stages 2 and 4 [3, 21, 63]. Based on our current data, however, a definitive conclusion whether the observed increase in capillary Aβ34 immunoreactivity in the earlier Braak stages indeed represents a histopathological correlate of elevated CSF Aβ34 levels detected in the recently published prodromal AD clinical sample [30] cannot be drawn at this point. On the one hand, we were not able to assess CSF Aβ34 levels in our post-mortem cohort. On the other hand, the post-mortem neuropathological sample was clinically divided only into two diagnostic categories, i.e. AD dementia and non-demented controls, the latter including both healthy elderly individuals and non-demented controls meeting the clinical criteria for a mild cognitive impairment. Another limitation of our study sample was the lack of younger individuals and age-matched controls of even earlier Braak stages (Braak stage 0) mainly due to the low prevalence and unavailability of this control group [37]. Analysis of such additional samples may be particularly helpful in clarifying the question whether pericyte mediated Aβ degradation occurs already at a very early stage in the absence of overt AD pathology, and whether it is upregulated in an age-dependent manner.

In our study, we provided substantial histological and biochemical evidence for an association of Aβ34 with brain pericytes. In vitro mechanistic assessments moreover revealed that pericytes can generate Aβ34 by uptake and degradation of extracellular Aβ40. Nevertheless, the results of these experiments do not exclude the possibility that Aβ34 is generated elsewhere, e.g. in neurons, and ultimately only taken up by pericytes for further degradation. As a major Aβ source and the primary cell type expressing BACE1 in the brain [5], neurons highly likely represent an alternative source of Aβ34 generation in the central nervous system. This assumption is also supported by a previous study reporting intraneuronal Aβ34 immunoreactivity [12]. The detection of capillary Aβ34 immunoreactivity, however, may in our opinion be better explained by an in situ production of Aβ34 in pericytes. Due to its short half-life and its susceptibility to further enzymatic degradation [11, 30], it appears rather unlikely that neuronal Aβ34 would reach the microvasculature in sufficient amounts in order to be taken up by brain pericytes. We rather propose that the more stable and more abundant Aβ isoforms like Aβ40 and possibly also Aβ42 which were previously shown to be drained along vascular clearance routes [59, 60] are taken up by pericytes and locally degraded to Aβ34 at the neurovascular unit. In line with this assumption, our mechanistic studies in human primary pericytes demonstrated that pericytes are fully capable of taking up and degrading Aβ40 to Aβ34. We did not specifically assess whether Aβ42 can also be degraded to Aβ34 by pericytes in vitro since the previously reported relative abundance of Aβ40 at the vasculature compared to Aβ42 [26, 53, 59] and our correlative analyses and findings in the human microvessels would rather be in line with Aβ40 being the major Aβ34 precursor in vivo. However, earlier studies illustrated that Aβ42 is also a substrate of BACE1 [19, 30] hence pericyte-associated Aβ34 could be generated via BACE1-mediated degradation of Aβ42. Both Aβ40 and Aβ42 fibrils were reported to induce pericyte death in vitro; nevertheless Aβ34 was shown not to aggregate even in high concentrations [11] and did not show any deleterious effects in our pericyte culture experiments (data not shown). Previously, Caillava et al. even suggested a protective role for this isoform since Aβ34 treatment lowered the apoptotic cell death in HEK cells in vitro [12]. Considering susceptibility of this isoform to further enzymatic degradation by metalloproteases [12, 30], we suggest that BACE-1 mediated degradation of Aβ40 or Aβ42 would enhance both enzymatic and vascular clearance by generating more accessible, benign and likely protective against apoptosis, isoform Aβ34.

The increase in Aβ34 immunoreactivity which we observed in our post-mortem sample may reflect a compensatory upregulation of vascular Aβ degradation in the early (preclinical or prodromal) stages of AD. As mentioned above, such an upregulation of Aβ34-related degradation clearance could provide an explanation for the observed increase in CSF Aβ34 levels in patients with prodromal AD [30]. As an alternative, elevated CSF Aβ34 levels might also be explained by an unspecific release of Aβ34 by degenerating cells, which in our case would be pericytes. We could not fully address with our cross-sectional correlative postmortem study design, whether the loss of Aβ34 immunoreactivity is caused by the physical loss of pericytes during disease progression. The decrease in Aβ34+ vessels appeared to be more pronounced than the decrease in the percentage of PDGFR-β + capillaries with advancing disease stages, suggesting in addition to the loss of pericytes, a functional impairment might have contributed to the observed findings. Nevertheless, the robust positive correlation between Aβ34 and PDGFR-β immunoreactivity in the brain and levels in our microvessel preparations support that at least partially an early loss of pericytes could be an important factor. In that regard, Aβ34 could also represent a sensitive candidate marker reflecting impairments in cerebrovascular pericyte function.

In the present study, we occasionally observed Aβ34 immunoreactivity in association with CAA in arterioles and large arterial vessels. However, only a limited number of individuals in our sample set showed CAA pathology, and capillary CAA (capCAA) was completely absent (Additional file 4). Wisniewski et al. previously reported presence of Aβ fibrils in cerebral vessels associated with pericytes [64]; due to a lack of capillary CAA cases we were unfortunately not able to assess the association between Aβ34 and aggregated Aβ forms in capillaries in our study. In case of arterial CAA, we speculate that Aβ34 immunoreactivity in these arteries might be the result of binding of this isoform to deposited vascular amyloid in arterial walls, e.g. upon failed vascular drainage. Alternatively, Aβ34 could also be locally generated in CAA-laden vessels, e.g. through the proteolytic cleavage of longer Aβ isoforms by BACE1 or MMPs [19, 25]. In support of this hypothesis, elevated levels of vascular BACE1 were previously described in CAA-laden arteries [7, 13, 16]. Further studies focusing on CAA and vascular amyloidosis in samples including capCAA will be required to obtain a better understanding of the origin of Aβ34 in CAA-affected arteries.

Pericytes have vital roles in vascular development, formation and maintenance of the blood brain barrier (BBB), blood flow regulation as well as in modulating immune reactions in the brain [1, 2, 4, 15, 29]. In addition, it has recently been shown that pericytes can take up extracellular Aβ42 via a LRP1/ APOE dependent mechanism in vitro [32]. Pericyte deficiency was shown to worsen AD pathology in transgenic animals with enhanced Aβ levels in the brain; conversely, pericyte implantation improved clearance of Aβ and reduced Aβ deposition [44, 55]. Given their location in the intramural periarterial drainage pathways and their ability to take up and degrade extracellular Aβ, pericytes have been implicated to have a role in Aβ clearance [17, 24]. We have shown, for the first time, an association of these cells with an important intermediate product of Aβ clearance. Our results are thus in line with the emerging role of pericytes in enzymatic Aβ degradation in human brain [32]. A failure of the pericyte-mediated Aβ degradation pathway could result in a progressive loss of vascular Aβ clearance, enhanced Aβ40 accumulation in the vasculature and consecutive impact on parenchymal Aβ clearance and deposition. The strong inverse association between Aβ34 immunoreactivity and tau pathology which we detected in our post-mortem sample suggests that this clearance pathway may have tremendous impact on neurodegenerative processes associated with AD.

## Conclusions

In the present study were able to show a unique association of the Aβ degradation intermediate Aβ34 with brain pericytes in capillaries of non-demented elderly individuals and patients with manifest AD dementia. The observed loss of pericytic Aβ34 in AD suggests an early failure of this novel Aβ clearance pathway at the neurovascular unit. Collectively, our findings strengthen the role of the vascular system in early AD and provide further support for a key role of brain pericytes in Aβ clearance and disease pathogenesis [30, 54].

## Supplementary information

**Additional file 1.** Primary antibodies used in immunofluorescence (IF), immunocytochemistry (ICC) and western blot (WB).

**Additional file 2.** Additional Aβ34-PDGFR-β immunostainings. ThioS staining. Vessel density quantification.

**Additional file 3.** Additional in vitro pericyte culture results.

**Additional file 4.** Cerebral Amyloid Angiopathy (CAA) quantification.

## Data Availability

The datasets used and/or analyzed during the current study available from the corresponding author on reasonable request.

## Abbreviations

AD: Alzheimer’s disease
ANOVA: Analysis of variance
APOE: Apolipoprotein E
APP: Amyloid precursor protein
Aβ: Amyloid-beta
BACE1: β-site APP-cleaving enzyme
CAA: Congophilic amyloid angiopathy
CERAD: Consortium to Establish a Registry for Alzheimer’s Disease
CSF: Cerebrospinal fluid
ELISA: Enzyme-linked immunosorbent assay
GFAP: Glial fibrillary acidic protein
HBSS: Hank’s balanced salt solution
HRP: Horseradish peroxidase
IPA3: 1,1′-Dithiodi-2-naphthtol
LRP1: Lipoprotein receptor-related protein
MCI: Mild cognitive impairment
MMP: Matrix metalloprotease
MSD: Meso scale discovery
NBB: Netherlands Brain Bank
NDCNTRL: Non-demented control
NFT: Neurofibrillary tangle
PBS: Phosphate-buffered saline
PDGFR-β: Platelet derived growth factor receptor beta
PFA: Paraformaldehyde
RAP: Receptor associated protein
