# Supplementary material for: The amyloid-β degradation intermediate Aβ34 is pericyte-associated and reduced in brain capillaries of patients with Alzheimer’s disease
Source: Acta Neuropathol Commun. 2019 Dec 3;7:194. doi: 10.1186/s40478-019-0846-8 (PMC6892233; doi:10.1186/s40478-019-0846-8)
Supplement: Supplementary file 1 — Additional file 1. Primary antibodies used in immunofluorescence (IF), immunocytochemistry (ICC) and western blot (WB). [file 40478_2019_846_MOESM1_ESM.pdf]

Additional File 1 - Primary antibodies used in immunofluorescence (IF), immunocytochemistry (ICC) and western blot (WB)

Target	Host	Clone	Source	Product number	Dilution*
Aβ34	Mouse	-	Multhaup Lab [29]	-	1:400
PDGFR-β	Goat	Polyclonal	R&D Systems	AF385	1:250 (IF), 1:200 (ICC), 1:2000 (WB)
Collagen IV	Rabbit	Polyclonal	Abcam	ab6586	1:500
CD31	Rabbit	Polyclonal	Abcam	ab32457	1:200
GFAP	Rabbit	Polyclonal	Sigma Aldrich	G9269	1:50
Amyloid	Mouse	W02	Sigma Aldrich	MABN10	1:800
Tau	Mouse	AT8	Thermofischer	MN1020	1:500
LRP1	Rabbit	EPR3724	Abcam	ab92544	1:200 (ICC), 1:2000 (WB)
BACE1	Rabbit	D10E5	Cell Signaling	5606T	1:200 (ICC), 1:1000 (WB)

* Dilutions were reported based on 1 mg/ml antibody stock solution
