# Supplementary material for: The amyloid-β degradation intermediate Aβ34 is pericyte-associated and reduced in brain capillaries of patients with Alzheimer’s disease
Source: Acta Neuropathol Commun. 2019 Dec 3;7:194. doi: 10.1186/s40478-019-0846-8 (PMC6892233; doi:10.1186/s40478-019-0846-8)
Supplement: Supplementary file 2 — Additional file 2. Additional Aβ34-PDGFR-β immunostainings. ThioS staining. Vessel density quantification. [file 40478_2019_846_MOESM2_ESM.pdf]

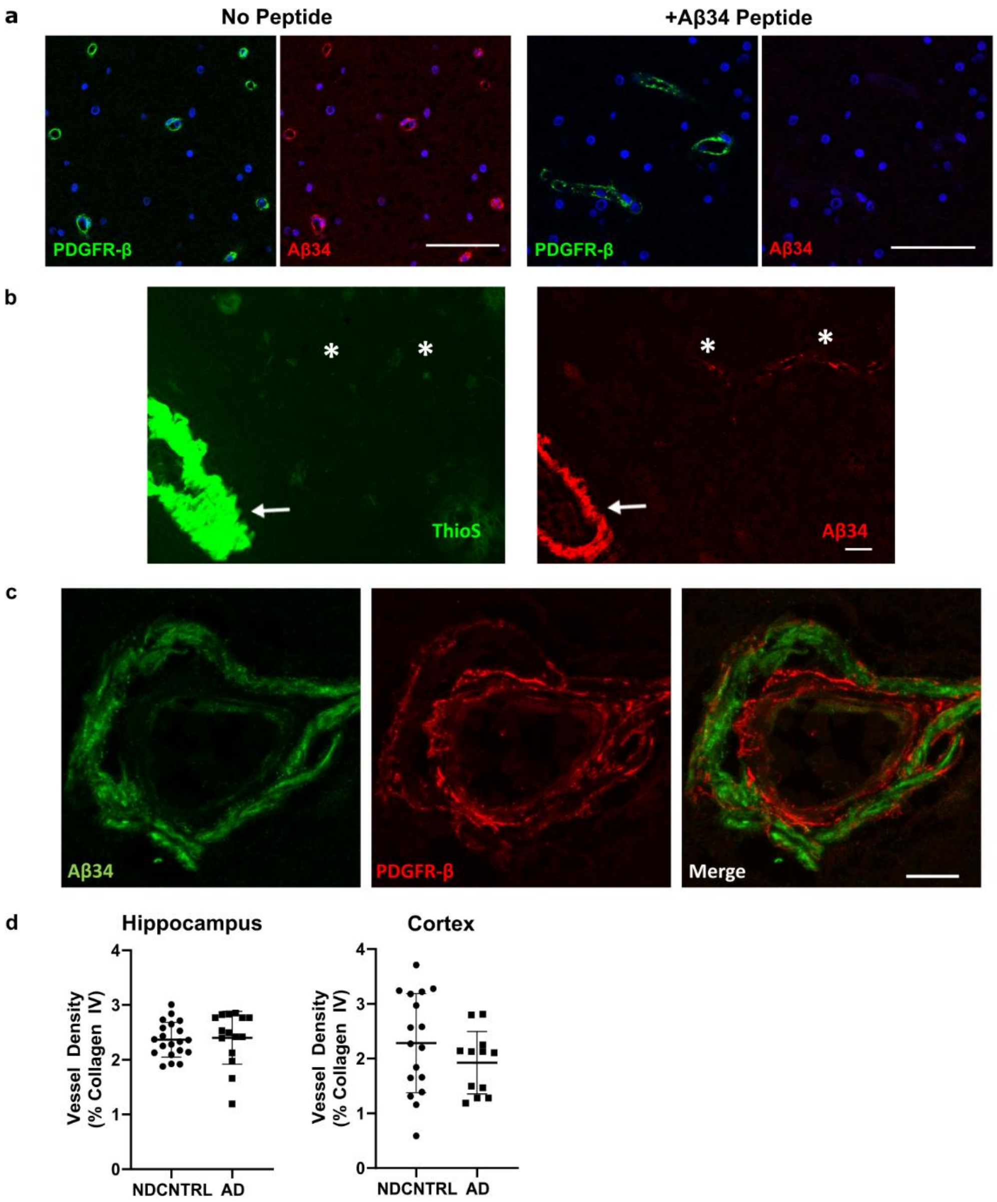

Additional File 2

a) Immunostaining of PDGFR-β (green), Aβ34 (red) and DAPI (blue) with or without the peptide competition. For the right panel, antibodies were pre-incubated with human recombinant Aβ34 peptide (5-fold more by weight) prior to primary antibody addition in immunofluorescence protocol. Pre-incubation with Aβ34 peptide led to complete loss of Aβ34 immunoreactivity supporting the specificity of the staining whereas PDGFR-β staining remained unchanged. (Scale bar 20 µm) **b)** ThioS and Aβ34 staining in consecutive brain sections. Infrequently detected artery with Aβ34 immunoreactivity (arrow) was also positive for ThioS staining. Conversely, more abundant capillary associated Aβ34 immunoreactivity (stars) was not detected with ThioS staining. **c)** Immunostaining of PDGFR-β (red) and Aβ34 in large artery located within the brain. Due to the size of the vessel, PDGFR-β immunostaining represents smooth muscle cell layer. Aβ34 immunoreactivity was detected colocalizing with CAA deposits. (Scale bar 20 µm) **d)** Quantification of capillary vessel density (assessed by area covered by Collagen IV immunostaining) shows no significant differences between non-demented controls (NDCNTRL) and AD patients (AD) in both hippocampus and cortex.
