# Supplementary material for: The amyloid-β degradation intermediate Aβ34 is pericyte-associated and reduced in brain capillaries of patients with Alzheimer’s disease
Source: Acta Neuropathol Commun. 2019 Dec 3;7:194. doi: 10.1186/s40478-019-0846-8 (PMC6892233; doi:10.1186/s40478-019-0846-8)
Supplement: Supplementary file 3 — Additional file 3. Additional in vitro pericyte culture results. [file 40478_2019_846_MOESM3_ESM.pdf]

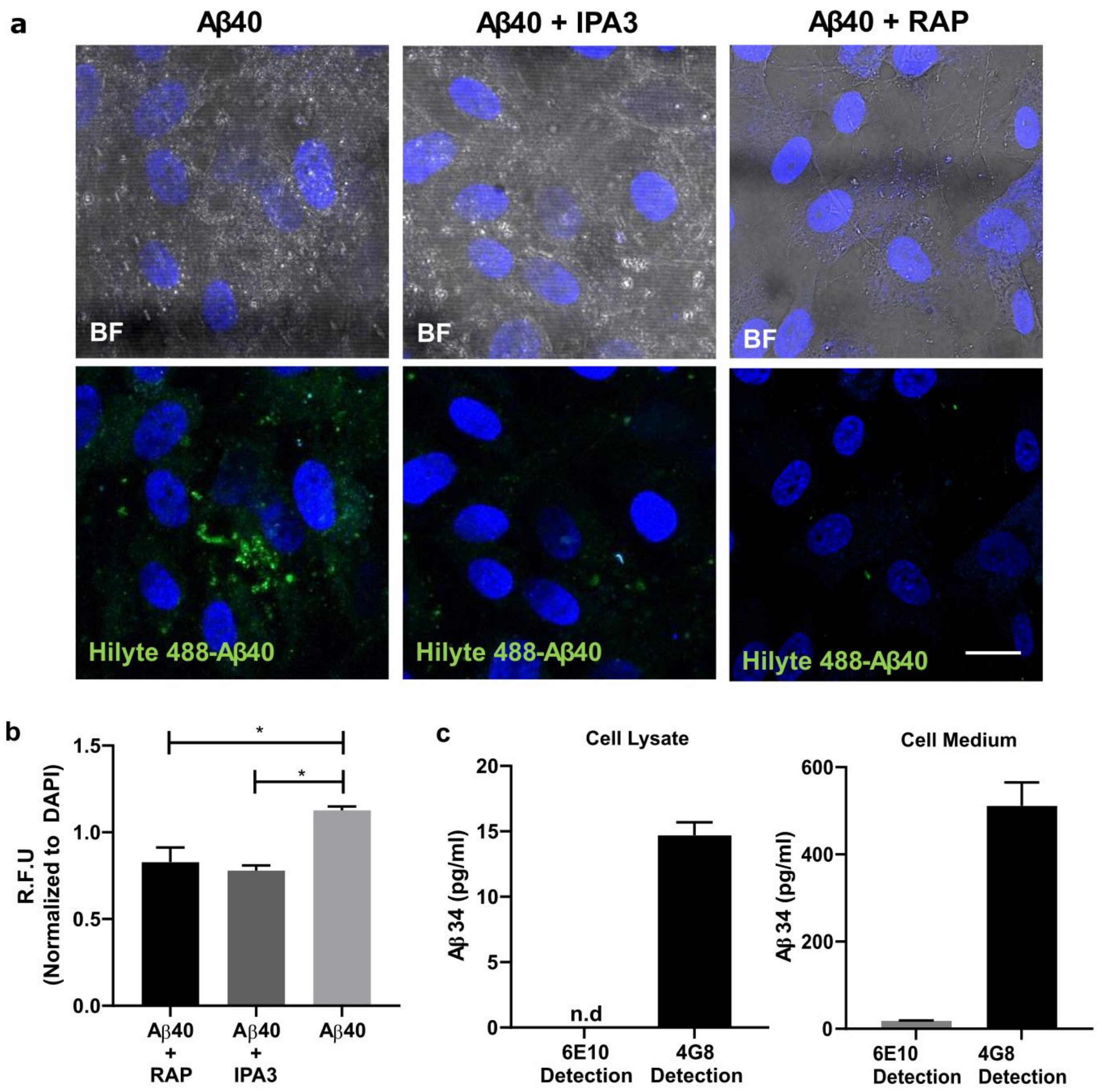

Additional File 3

a) Immunocytochemistry-based assay of fluorescently-labeled Aβ40 (Hilyte 488-Aβ40) uptake of pericytes upon IPA3 or RAP treatment. (Scale bar 10 µm). **b)** 96-well plate assay of fluorescently-labeled Aβ40 uptake of pericytes upon IPA3 or RAP treatment. **c)** Treatment of pericytes with mouse Aβ40 and detection of Aβ34 with a human specific (6E10) or mid-domain (4G8) antibody which can detect both mouse and human Aβ.
