# Supplementary material for: The amyloid-β degradation intermediate Aβ34 is pericyte-associated and reduced in brain capillaries of patients with Alzheimer’s disease
Source: Acta Neuropathol Commun. 2019 Dec 3;7:194. doi: 10.1186/s40478-019-0846-8 (PMC6892233; doi:10.1186/s40478-019-0846-8)
Supplement: Supplementary file 4 — Additional file 4. Cerebral Amyloid Angiopathy (CAA) quantification. [file 40478_2019_846_MOESM4_ESM.pdf]

Additional File 4 - Cerebral Amyloid Angiopathy (CAA) quantification

	Brain Region: Hippocampus		
**Braak Stage**	**I-II**	**III-IV**	**V-VI**
**CAA Score[a]**	Grade 0 (3)<br>Grade 1 (3)<br>Grade 2 (1)<br>Grade 3 (0)<br>Grade 4 (0)	Grade 0 (4)<br>Grade 1 (0)<br>Grade 2 (5)<br>Grade 3 (1)<br>Grade 4 (0)	Grade 0 (4)<br>Grade 1 (0)<br>Grade 2 (0)<br>Grade 3 (2)<br>Grade 4 (2)
	**Brain Region: Cortex**		
**Braak Stage**	**I-II**	**III-IV**	**V-VI**
**CAA Score**	Grade 0 (10)<br>Grade 1 (1)<br>Grade 2 (1)<br>Grade 3 (0)<br>Grade 4 (0)	Grade 0 (7)<br>Grade 1 (2)<br>Grade 2 (4)<br>Grade 3 (0)<br>Grade 4 (0)	Grade 0 (5)<br>Grade 1 (0)<br>Grade 2 (3)<br>Grade 3 (2)<br>Grade 4 (0)

a: Capillary CAA was absent in the sample set. CAA scores show the prevalence and severity of CAA-laden arteries. CAA Score: Grade 0 = none, Grade 1 = mild, Grade 2 = moderate, Grade 3 = severe, Grade 4 = very severe (Adapted from Arvanitakis Z, Leurgans SE, Wang Z, Wilson RS, Bennett DA, Schneider JA (2011) Cerebral amyloid angiopathy pathology and cognitive domains in older persons. Annals of Neurology 69:320–327. doi: 10.1002/ana.22112)
